# On the Evaluation of a Novel Hypoxic 3D Pancreatic Cancer Model as a Tool for Radiotherapy Treatment Screening

**DOI:** 10.3390/cancers13236080

**Published:** 2021-12-02

**Authors:** Gabrielle Wishart, Priyanka Gupta, Andrew Nisbet, Giuseppe Schettino, Eirini Velliou

**Affiliations:** 1 Bioprocess and Biochemical Engineering Group (BioProChem), Department of Chemical and Process Engineering, University of Surrey, Guildford GU2 7XH, UK; g.wishart@surrey.ac.uk (G.W.); priyanka.g.gupta@ucl.ac.uk (P.G.); 2 Department of Physics, University of Surrey, Guildford GU2 7XH, UK; giuseppe.schettino@surrey.ac.uk; 3 Centre for 3D Models of Health and Disease, Department of Targeted Intervention, Division of Surgery and Interventional Science, University College London (UCL), London W1W 7TY, UK; 4 Department of Medical Physics and Biomedical Engineering, University College London (UCL), London WC1E 6BT, UK; andrew.nisbet@ucl.ac.uk; 5 National Physical Laboratory, Teddington TW11 0LW, UK

**Keywords:** pancreatic cancer, tissue engineering, tumour microenvironment (TME), treatment resistance, radiotherapy, radiation, radioprotection, hypoxia, polyurethane scaffolds, 3D cell culture, extracellular matrix (ECM), HIF-1a, PANC-1

## Abstract

**Simple Summary:**

Pancreatic cancer challenges global health with non-specific symptoms, devastatingly low survival rates, and high treatment resistance profiles. Tissue engineering is advancing to facilitate animal free tissue biomimicry, allowing the replication of tumour tissue specific hallmarks of pancreatic cancer that challenge modern treatments. Here, we report the development and characterisation of a low oxygen (hypoxic) 3D polyurethane scaffold system for long-term analysis of radiation responses. This finely tuned platform more accurately recapitulates bio-physical, bio-chemical, and structural-bio-mechanical in vivo tissue niches as well as tumour hypoxia. The latter is a treatment-limiting feature for radiotherapy, allowing the system to streamline the transition of clinical testing from bench to bedside.

**Abstract:**

Tissue engineering is evolving to mimic intricate ecosystems of tumour microenvironments (TME) to more readily map realistic in vivo niches of cancerous tissues. Such advanced cancer tissue models enable more accurate preclinical assessment of treatment strategies. Pancreatic cancer is a dangerous disease with high treatment resistance that is directly associated with a highly complex TME. More specifically, the pancreatic cancer TME includes (i) complex structure and complex extracellular matrix (ECM) protein composition; (ii) diverse cell populations (e.g., stellate cells), cancer associated fibroblasts, endothelial cells, which interact with the cancer cells and promote resistance to treatment and metastasis; (iii) accumulation of high amounts of (ECM), which leads to the creation of a fibrotic/desmoplastic reaction around the tumour; and (iv) heterogeneous environmental gradients such as hypoxia, which result from vessel collapse and stiffness increase in the fibrotic/desmoplastic area of the TME. These unique hallmarks are not effectively recapitulated in traditional preclinical research despite radiotherapeutic resistance being largely connected to them. Herein, we investigate, for the first time, the impact of in vitro hypoxia (5% O_2_) on the radiotherapy treatment response of pancreatic cancer cells (PANC-1) in a novel polymer (polyurethane) based highly macroporous scaffold that was surface modified with proteins (fibronectin) for ECM mimicry. More specifically, PANC-1 cells were seeded in fibronectin coated macroporous scaffolds and were cultured for four weeks in in vitro normoxia (21% O_2_), followed by a two day exposure to either in vitro hypoxia (5% O_2_) or maintenance in in vitro normoxia. Thereafter, in situ post-radiation monitoring (one day, three days, seven days post-irradiation) of the 3D cell cultures took place via quantification of (i) live/dead and apoptotic profiles and (ii) ECM (collagen-I) and HIF-1a secretion by the cancer cells. Our results showed increased post-radiation viability, reduced apoptosis, and increased collagen-I and HIF-1a secretion in in vitro hypoxia compared to normoxic cultures, revealing hypoxia-induced radioprotection. Overall, this study employed a low cost, animal free model enabling (i) the possibility of long-term in vitro hypoxic 3D cell culture for pancreatic cancer, and (ii) in vitro hypoxia associated PDAC radio-protection development. Our novel platform for radiation treatment screening can be used for long-term in vitro post-treatment observations as well as for fractionated radiotherapy treatment.

## 1. Introduction

Pancreatic ductal adenocarcinoma (PDAC) is a cancer of the duct cells of the pancreas. Most commonly found in the head of the pancreas, this disease is the most common form of pancreatic cancer (96% of pancreatic cancers present as PDAC) as opposed to more rare pancreatic cancers (e.g., neoendocrine tumours) [1,2]. PDAC challenges global health with devastatingly low 5-year survival rates (10%) compared to other cancers (e.g., breast cancer (90%), prostate cancer (89%), and melanoma of the skin (93%)) [1,2]. Increasing incidence rates, non-specific symptoms, and late diagnosis of this disease elucidate high mortality rates [1,2,3]. At diagnosis, a small fraction of patients (20%) are eligible for curative surgery due to late detection and high metastatic occurrence [1]. Chemotherapeutics such as Gemcitabine, Capecitabine, and FOLFIRINOX are suggested for consideration for first line, adjuvant, or for metastatic PDAC treatment [3]. Radiotherapy for PDAC is suggested for consideration as an adjuvant therapy. However, this option has national variations in treatment recommendations due to limited data [2] and controversial European clinical trials [4,5]. A pooled analysis of 955 PDAC patients treated with adjuvant chemo-radiotherapy showed improved overall survival of patients compared to chemotherapy alone [6]. Thus, the role of radiotherapy for pancreatic cancer is largely debated and thought to be still evolving [2,4,5,6,7,8].

Pancreatic cancer is notoriously resistant to current chemotherapy and radiotherapy treatments due to an extremely complex tumour microenvironment [9,10,11,12]. The PDAC tumour microenvironment (TME) is a distinct in vivo milieu of cellular, biochemical, and biomechanical features that encompass distinct fingerprints that promote tumour cell survival, migration, and resistance to treatments [9,13,14,15,16,17]. These unique hallmarks include (i) complex structure and complex extracellular matrix (ECM) protein composition; (ii) diverse cell populations (e.g., stellate cells, cancer associated fibroblasts, endothelial cells, immune cells) that interact with the cancer cells and promote resistance to treatment and metastasis; (iii) accumulation of high amounts of ECM, which leads to the creation of a fibrotic/desmoplastic reaction around the tumour; and (iv) heterogeneous environmental gradients such as hypoxia, which result from vessel collapse and stiffness increase in the fibrotic/desmoplastic area of the TME. More specifically, chaotic cancer cell growth activates pancreatic stellate cell secretion of extracellular matrix (ECM) proteins, known as the desmoplastic reaction [9,14,15,18]. This large increase in ECM protein deposit (i.e., collagen, fibronectin, and laminin) and tumour stiffness, along with very high cancer cell growth, influences pro-survival characteristics and causes intra-tumoral blood vessel disruption and collapse, consequently impairing (chemotherapeutic) drug delivery (resulting in chemo-resistance) and causing heterogeneous expanses of low oxygen gradients (hypoxia) [9,13,14,15,16,17,18]. Tumour hypoxia influences instrumental changes to the TME micromilieu promoting cancer growth, metastasis, invasion, and resistance to radiotherapy, [17,19,20,21,22]. More specifically, over 50 years of research describes the reduced effectiveness of radiation (up to a factor of 2.5–3) in the absence of oxygen, as explained by the oxygenation fixation hypothesis (OFH) [17,22]. Attempts to target the hypoxic hallmark are emerging [23,24] with limited clinical progress. Unrealistic pre-clinical models that fail to recapitulate treatment-limiting hallmarks such as hypoxia are impeding treatment success [25]. Thus, the need to understand and recapitulate in vitro this complicated ecosystem of diverse cellular and non-cellular components that create the unique PDAC TME niche is a matter of clinical relevance for the optimisation of treatments such as radiotherapy for PDAC.

Preclinical treatment screening has traditionally utilised (a) 2D in vitro systems and (b) animal models. 2D cell culture systems are a fast cost-effective gold standard for therapy testing in vitro, however, this method does not incorporate realistic TME hallmarks such as microarchitecture, stiffness, spatial orientation, cell–cell and cell–extracellular matrix protein interactions, and environmental gradients of the TME that are associated with radiation response (i.e., hypoxia) [25,26,27,28,29,30,31]. In the literature, the exposure of 2D cell culture systems (tissue culture flasks or micro-plates) to hypoxia in gas controlled chambers has been utilised to identify hypoxia-associated genes [32], to evaluate hypoxic sensitisers [33], investigate damage repair pathways, and improve understanding of cell adaptions to hypoxia [34,35]. Typically 72 h of hypoxic exposure in 2D are described as chronic hypoxic conditions, a timeframe that is not in-line with heterogeneous oxygen expanses in vivo [34]. Animal models support more realistic microarchitecture, stiffness and cell–TME crosstalk compared to 2D cell culture systems [25,27,28,36]. These models are the most widely used for drug and radiotherapy pre-clinical testing, and can be utilised to study (i) the influence of hypoxia on metastatic disease progression [37]; (ii) hypoxia activated pro-drugs [38]; and (iii) radio-sensitisers [39]. However, animal models are expensive, complex, not always reproducible, challenging to use, and they also raise ethical concerns. Moreover, discrepancies in physiology, size and genetics limit the reproducibility of the studies and can result in translational errors in the clinic [25,27,28,36,40].

As a result, there is growing interest in the development and use of 3D cancer in vitro models for the replacement of 2D in vitro models and reduction in the use of animal models, the latter of which is an important objective in terms of the 3R framework (replace, reduction, and refinement of animals) [41,42]. 3D models for pre-clinical treatment testing are emerging to more realistically recapitulate TMEs, and more specifically TME hallmarks that are associated with treatment resistance. TME mimicry encourages the cells to employ behavioural and physiological characteristics that more similarly emulate realistic in vivo properties to allow for a more streamlined transition of clinical treatment testing from laboratory bench to patient bedside [25,27,28,43,44]. 3D PDAC models have emerged to mimic in vivo niches, these include (i) spheroids [45,46]; (ii) hydrogels [47]; and (iii) polymeric scaffolds [26,27,28,43]. With respect to radiotherapy in 3D PDAC in vitro models, spheroids and polymeric scaffolds as tools specifically for radiotherapy screening have started to emerge [25] and are required to test new modalities [48]. 

Spheroid models are simple cell clusters/aggregates in suspension, they have featured in research articles reporting radiation response studies and investigating radiosensitiser potential for pancreatic cancer. For example, Al-Ramadan et al. (2018) utilised spheroids to identify radiation dose (0–6 Gy) dependent sensitivity in the pancreatic neuroendocrine cell line BON-1 seven days post treatment via apoptosis induction [49]. Moreover, Al-Assar et al. (2014) reported that the co-culture of the pancreatic cancer cells (PANC-1) and stellate cells (PSC) enhanced radio-resistance (0–6 Gy) in sphere models (eight days) similar to their xenograft models (60 days) [50]. Furthermore, Hehlgans et al. (2009) identified Caveolin-1 and TAE226 as potential radiosensitisers (in radiotherapy doses of 0–6 Gy) for the pancreatic cancer cell line MiaPacCa2 in a spheroid system [51,52]. Moreover, PDAC spheroids are emerging as platforms to test new modalities such as proton therapy and boron neutron capture therapy [53,54]. Overall, spheroid models can be considered an advanced platform supporting more realistic 3D cellular interactions compared to traditional 2D cell cultures for radiation response studies, however, these models lack robust porosity, ECM controlled composition, and mechanical stability [25,27,28,36]. As a result, long-term radiation studies can be challenging.

Polymeric scaffolds have featured in very few research articles reporting radiation response studies for PDAC. These scaffolds are made of biocompatible polymers and have controlled stiffness and internal architecture (porosity or fibrous internal organisation). We have previously reported the long-term culture (35 days) of PDAC cells lines (PANC-1, AsPC-1, BxPC-3) in highly macroporous polyurethane (PU) polymeric scaffolds. In our previous work, we surface modified our scaffolds with fibronectin. Fibronectin is one of the most abundant proteins present in the ECM of the pancreatic cancer tumour microenvironment [55]. Therefore, the scaffolds were surface modified with fibronectin to enhance ECM mimicry and improve cell–cell and cell–matrix interactions. Indeed fibronectin modified scaffolds have demonstrated dense spatial cellular masses, collagen-I production from the cancer cells, and environmental (hypoxic) gradients that followed an in vivo-like trends. In contrast, cells in non-coated scaffolds did not have a physiological behaviour [27]. Furthermore, we have performed chemotherapy (with 10 μm, 50 μm, 100 μm GEM), radiotherapy (with 0 Gy, 2 Gy, 6 Gy, 8 Gy), and chemoradiotherapy (with 10 μm GEM and radiotherapy of 6 Gy) screening on those fibronectin modified polyurethane (PU) scaffolds [28]. More specifically, we have reported dose dependent chemotherapy and radiotherapy viability drop and apoptosis induction after short-term (one day) and long-term (17 days) PANC-1 cell culture, with chemoradiotherapy being more efficient than single radiation doses. Similar trends were seen at both short- and long-term, with 17 days being the longest reported in vitro post-treatment timeframe [28]. This is the first long-term scaffold based platform employing radiation treatment screening for pancreatic cancer [28].

To the best of our knowledge, the combination of hypoxic exposure and radiation modalities have not been tested in 3D scaffold models for PDAC. Therefore, the aim of this work was to investigate, for the first time, the effect of hypoxia on the response of pancreatic cancer cells to radiotherapy, in our previously developed highly porous, fibronectin coated 3D polyurethane scaffold. Overall, we report (i) a novel long-term in vitro hypoxic 3D cell culture system for pancreatic cancer; and (ii) the development of in vitro hypoxia associated PDAC radio-protection. Our novel platform for radiation treatment screening can be used for long-term in vitro post-treatment observations as well as for fractionated radiotherapy treatment; the latter being standard practice in the clinic.

## 2. Materials and Methods

### 2.1. Scaffold Fabrication and Surface Modification

**Figure A1:**
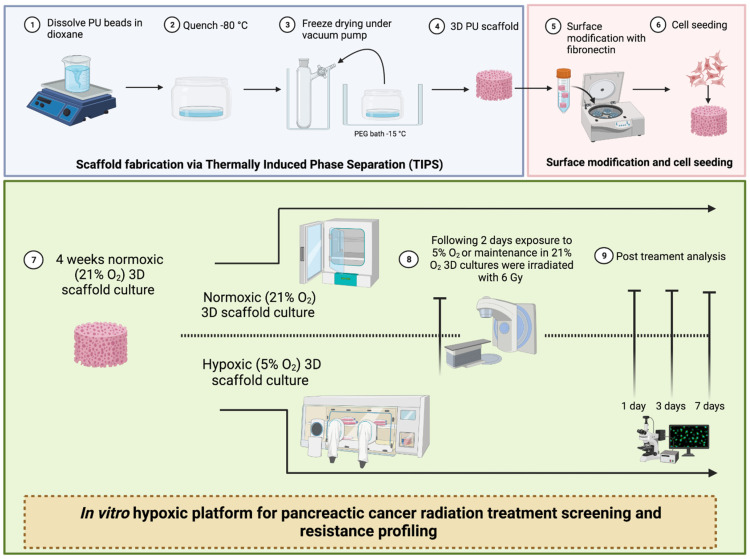
Experimental design. Including scaffold fabrication, surface modification, and cell seeding. Created with BioRender.com.

Polymeric scaffolds were fabricated via the thermally induced phase separation (TIPS) method as reported previously [27,28,56] (Appendix A Figure A1). More specifically, 3 g of polyurethane (PU) beads (Noveon, Oudergem, Belgium) was dissolved in 60 mL dioxane (5% *w*/*v*) (99.8% anhydrous pure, Sigma-Aldrich, Merck, Gillingham, UK) for 48 h before the solution was quenched at −80 °C for 3 h. The solvent was removed via freeze-drying in a polyethylene glycol (PEG) bath at −15 °C under 0.01 mbar vacuum pressure for 72 h. Scaffolds were snap frozen in liquid nitrogen following immediate cutting into 5 × 5 × 5 mm^3^ cubes. Thereafter, the scaffolds were sterilised via 70% ethanol submersion (3 h) and UV ray exposure (1 h). The average pore size of the scaffold was 100–150 μm, the porosity was 85–90%, and the elastic modulus 20 ± 2 kPa, with stiffness similar to ex vivo high stiffness diseased PDAC tissue, as previously reported [27,28,56,57,58,59]. Thereafter, the scaffolds were surface modified (adsorption) to enable coating with fibronectin (i.e., an ECM protein extensively present in the PDAC TME for ECM biomimicry). As mentioned in the introduction, we have previously reported physiological behaviour of PDAC cells in the presence of fibronectin (dense cell aggregates, collagen-I secretion by the cancer cells, and realistic environmental gradients) compared to sparser cell organisation and no collagen production in uncoated scaffolds [27]. Briefly, for surface modification with fibronectin, the scaffolds were centrifuged in phosphate buffered saline (PBS, Sigma-Aldrich, Merck, UK) for 10 min at 2500 rpm, then centrifuged in fibronectin solution (25 μg mL^−1^) for 20 min at 2000 rpm, before finally being centrifuged in PBS for 10 min at 1500 rpm.

### 2.2. Cell Culture

The 3D cell culture (in the scaffolds) was accomplished as described previously [27]. More specifically, human pancreatic adenocarcinoma cells (PANC-1) (ATCC) were initially expanded in 2D flasks, in Dulbecco’s Modified Eagle’s Medium (DMEM) with high glucose (Sigma-Aldrich, Merck, UK) supplemented with 10% foetal bovine serum (Fisher Scientific, Loughborough, UK), 1% penicillin/streptomycin (Fisher Scientific, UK), and 2 mM L-glutamine (Sigma-Aldrich, Merck UK) in 37 °C with 21% O_2_ and 5% CO_2_. PANC-1 cells were passaged when 80–90% confluency was reached, until the appropriate cell number for the 3D experiments was obtained. Thereafter, 0.5 × 10^6^ PANC-1 cells were seeded per scaffold (re-suspended in 30 μL of cell culture media) and placed in 24-well plates. Thereafter, the scaffolds were placed in an incubator for 1 h to ensure adherence. Therein, 1.5 mL of cell culture media was added to each well, this was replaced every two days and each 24-well plate was replaced after one week to avoid cell egress from scaffolds and cell confluency on the bottom of each well. Incubation of all scaffolds took place in a humidified incubator at 37 °C with 21% O_2_ and 5% CO_2_ (in vitro normoxia) for 28 days (four weeks). Thereafter, half of the scaffolds were moved to in vitro hypoxic conditions at 37 °C with 5% O_2_ and 5% CO_2_ in a Ruskinn InvivO2 300 workstation (Baker Ruskinn, Ltd., Bridgend, UK) for a two day pre-treatment exposure to low oxygen. Post treatment analysis took place at one day, three days, and seven days post treatment in both (i) in vitro normoxic (21% O_2_) and (ii) in vitro hypoxic (5% O_2_) culture conditions.

### 2.3. Radiation Treatment

Radiation treatments were performed with a clinical 250 kVp X-ray irradiator, Xstrahl 300 (Xstrahl, Camberley, UK) at the Royal Surrey County Hospital NHS Foundation Trust (Calibrated against a previously published code of practice [60]). More specifically, at four weeks of culture of normoxic culture (21% O_2_) and after two day pre-treatment exposure to either in vitro hypoxia (5% O_2_) or normoxia maintenance, the scaffolds were irradiated with 6 Gy based on previous results and protocols for radiotherapy treatment in normoxia [28]. A square field applicator of 15 × 15 cm was placed 3 cm above the plate surfaced, which was placed on an epoxy resin water equivalent phantom (30 × 30 × 10 cm) in order to ensure a uniform radiation field with known radiation back scattering conditions. Control scaffolds were used to account for cellular stress during transportation from the University of Surrey to the Royal Surrey County Hospital. Thereafter, hypoxic scaffolds were placed back in 5% O_2_, normoxic scaffolds were placed back at 21% O_2_, and all scaffolds were analysed at one day, three days, and seven days post-treatment. 

### 2.4. Live/Dead Analysis via Confocal Imaging 

The distribution of live and dead cells within scaffolds for all conditions under study (treated and untreated for both normoxic and hypoxic scaffolds) was evaluated via the Live/Dead Viability/Cytotoxicity Kit (Molecular Probes, Thermo Scientific, Loughborough, UK). More specifically, scaffolds were snap frozen at time points of one day, three days, and seven days post-treatment in liquid nitrogen for 20 min and preserved thereafter at −80 °C as previously described [26,27,28,36]. Thereafter, the scaffolds were sectioned and stained with 2 μm of Calcien-AM (4 mM stock) and 4 μm of Ethidium Homodimer (2 mM stock) and incubated at 37 °C for 1 h. Thereafter, the samples were washed twice with PBS and visualised using a Nikon Ti-Eclipse inverted confocal microscope (Nikon Instruments, Amstelveen, The Netherlands).

### 2.5. Caspase 3/7 Analysis via Confocal Imaging

The distribution of apoptotic cells within scaffolds for all conditions under study was evaluated via Caspase 3/7 analysis. More specifically, scaffolds were snap frozen at time of points one day, three days, and seven days post-treatment in liquid nitrogen for 20 min and preserved thereafter at −80 °C. The scaffolds were sectioned and stained with Cell Event Caspase 3/7 green detection reagent (Fisher Scientific, UK) and DAPI (Fisher Scientific, UK) for 1 h at 37 °C. Thereafter, the samples were washed with PBS and visualised using a Nikon Ti-Eclipse inverted confocal microscope (Nikon Instruments, Amstelveen, The Netherlands).

### 2.6. Immunofluorescence Assay

Immunofluorescence staining took place to characterise collagen-I (Abcam, Cambridge, UK), and HIF-1a (Abcam, UK) spatial secretion in all conditions under study at various time points. More specifically, scaffolds were snap frozen at one day, three days, and seven days post-treatment in liquid nitrogen for 20 min and preserved thereafter at −80 °C prior to immunofluorescence staining. Scaffolds were sectioned and fixed for 4 h in 4% *w*/*v* paraformaldehyde (Sigma-Aldrich, Merck, UK). Thereafter, scaffold sections were permeabilised for 2 h with 0.1% Triton-X solution (Sigma-Aldrich, Merck, UK), followed by blocking with 10% donkey serum (Abcam, UK) for 3 h. Primary antibody staining was carried out overnight, followed by overnight secondary antibody staining including DAPI (Fisher Scientific, UK). Primary (Mouse HIF-1a Abcam, UK) and secondary (Alexa Flour 647 donkey anti-mouse IgG (H+L)) antibodies for HIF-1a were diluted in 1% *w*/*v* bovine serum albumin (Sigma-Aldrich, Merck, UK). 

### 2.7. Confocal Laser Scanning Microscopy

Live/Dead, Caspase 3/7 analysis, and immunofluorescence (Section 2.5, Section 2.6 and Section 2.7) were imaged on a Nikon Ti-Eclipse inverted confocal microscope (Nikon Instruments, Amstelveen, The Netherlands) and processed with the NIS-Elements software, using 405, 488, 555, 604, 647 lasers for DAPI (blue), Calcein AM and Caspase 3/7 (green), collagen-I (yellow) and Ethidium Homodimer and HIF-1a (red) staining, respectively. Images were captured at a 10× objective and 10 μm Z-stack distance. Multiple scaffolds, scaffold sections, and scaffold areas were imaged for each condition under study to ensure reproducibility. The images presented here are representative images from each condition.

### 2.8. Image Analysis

ImageJ^®^ software (Wayne Rasband, NIH, Bethesda, MD, USA) was utilised to quantify spatial characterisation of (i) Live/Dead; (ii) Caspase 3/7; (iii) collagen-I; (iv) HIF-1a; and (v) DAPI positive areas vs. negative areas. Multiple scaffolds (*n* = 3), scaffold sections (*n* = 3), and scaffold areas (*n* = 2) were analysed per condition to ensure reproducibility of results. The bars in each bar graph of the results represent averages of percentage areas of each fluorescence channel.

### 2.9. Statistical Analysis and Data Analysis

Graph Pad Prism^®^ (GraphPad Software, San Diego, CA, USA) was utilised to determine statistical significance (*p* < 0.05). Analysis of variance (ANOVA), followed by Tukey’s multiple comparison test were employed. Standard error of the mean was used to determine error bars in the bar graphs. Where data were normalised with respect to the control, the following equation was employed: % Caspase 3/7 area = ((treatment positive area)/(control positive area)) × 100.

## 3. Results

### 3.1. Evaluation of the Effect of Radiation Treatment on PANC-1 Cells in 3D Scaffolds

Following four weeks of culturing pancreatic cancer cells in 21% O_2_ in our highly porous polymer fibronectin coated scaffolds (i.e., at a timeframe that we have previously shown) allowed for the formation of PDAC physiological features such as dense spatial cell aggregates, metabolic gradients, and secretion of collagen-I by the PDAC cells [24]. The 3D scaffolds were either maintained in 21% O_2_ or were exposed to 5% O_2_ for two days. Thereafter, radiation treatment (6 Gy) took place followed by post-treatment monitoring of (i) the spatial secretion of the hypoxic biomarker HIF-1a in the 3D scaffolds; (ii) the cell viability and cell apoptosis in the 3D scaffolds; and (iii) the secretion of collagen-I by the cancer cells in the 3D scaffolds.

### 3.2. Post-Radiation Monitoring of the Hypoxic Biomarker HIF-1a in the 3D Scaffolds

**Figure 1:**
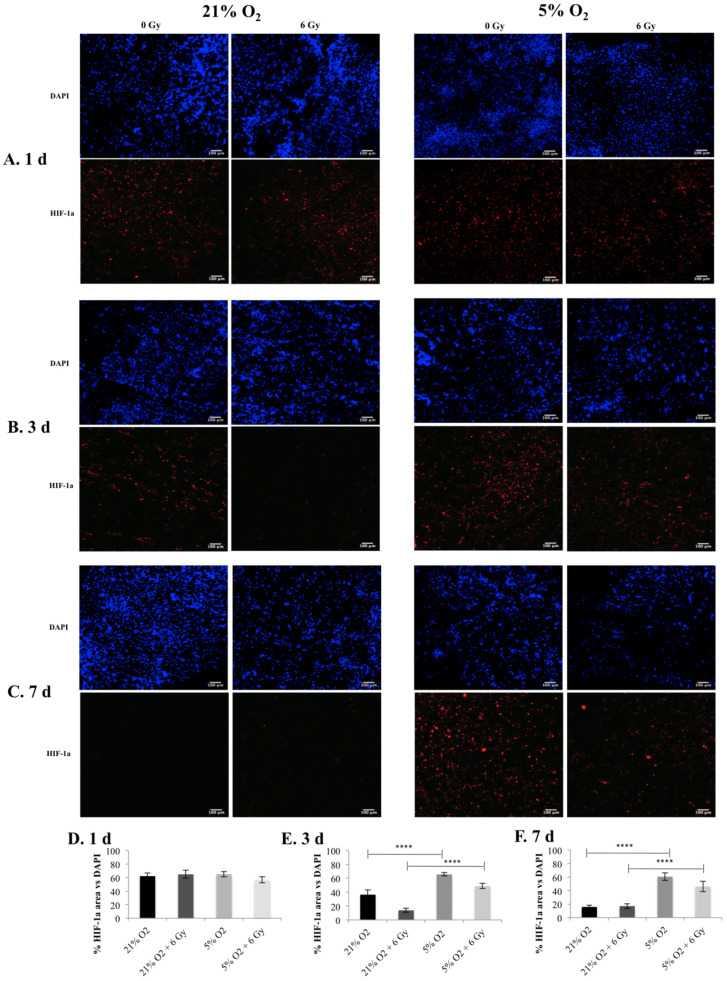
HIF-1a expression following radiotherapy treatment (6 Gy) in 3D scaffolds for 21% O_2_ and 5% O_2_: (**A**–**C**) Representative images of scaffold sections for HIF-1a (red) staining, counterstained with DAPI (blue) at 1 day, 3 days, and 7 days post radiation treatment. (**D**–**F**) Equivalent image analysis based quantification of the percentage of HIF-1a positive image areas over total cell (blue) image areas (DAPI) for 1 day (**D**), 3 days (**E**), and 7 days (**F**) post radiation treatment. Multiple scaffolds (3), scaffold sections (3), and images (2) were analysed, mean values are presented. (**** = *p* < 0.0001).

The biomarker hypoxia-inducible factor, HIF-1a was monitored for one, three, and seven days post-radiation in the 3D scaffolds, this hypoxic biomarker is hyper-expressed in PDAC in comparison to healthy tissue of the pancreas [61]. As one of the major hypoxia induced intracellular signalling pathways, the HIF-1a pathway is associated with pro-survival, metastasis, and resistance to therapy [17]. Figure 1 summarises confocal images of scaffold sections showing the HIF-1a spatial distribution along with a quantitative assessment of the percentage of HIF-1a positive areas in multiple images taken from multiple sections of multiple scaffolds. As can be seen in Figure 1, a significantly higher HIF-1a secretion in the 3D scaffolds was observed at 5% O_2_ for both treated and untreated scaffolds compared to 21% O_2_ for days 3 and 7 post-radiotherapy treatment, while no differences were observed between different oxygen conditions one day post-treatment.

### 3.3. Post-Radiation Monitoring of Cell Viability and Apoptosis in the 3D Scaffolds

The cell viability and the cell apoptosis in the 3D scaffolds were monitored for one, three, and seven days post-radiation with Live/Dead and Caspase 3/7 staining respectively.

**Figure 2:**
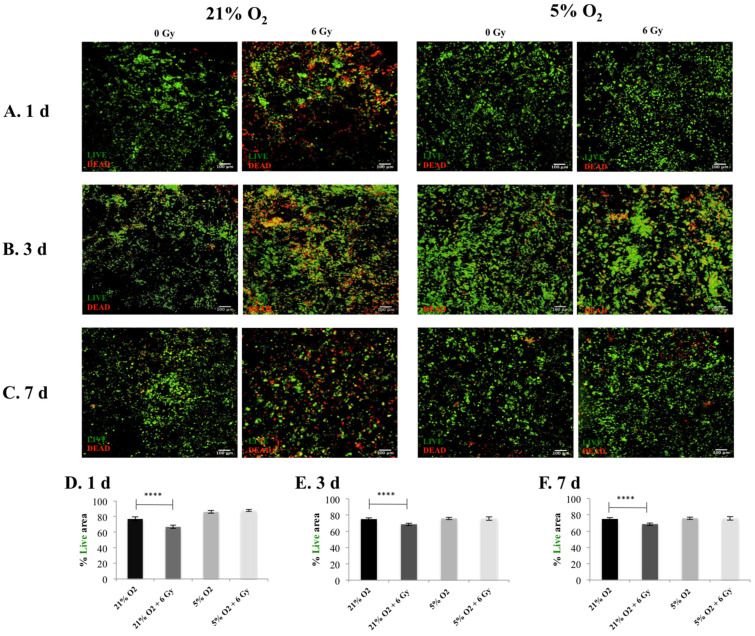
Cell viability (Live/Dead staining) following radiotherapy treatment (6 Gy) in 3D scaffolds for 21% O_2_ and 5% O_2_: (**A**–**C**) Representative images of scaffold sections for Live (green)/Dead (red) staining, 1 day, 3 days, and 7 days post-treatment and (**D**–**F**) equivalent image analysis based quantification of the percentage of Live (green) image areas for (**A**–**C**). Multiple scaffolds (3), scaffold sections (3), and images (2) were analysed, mean values are presented. (**** = *p* < 0.0001).

**Figure 3:**
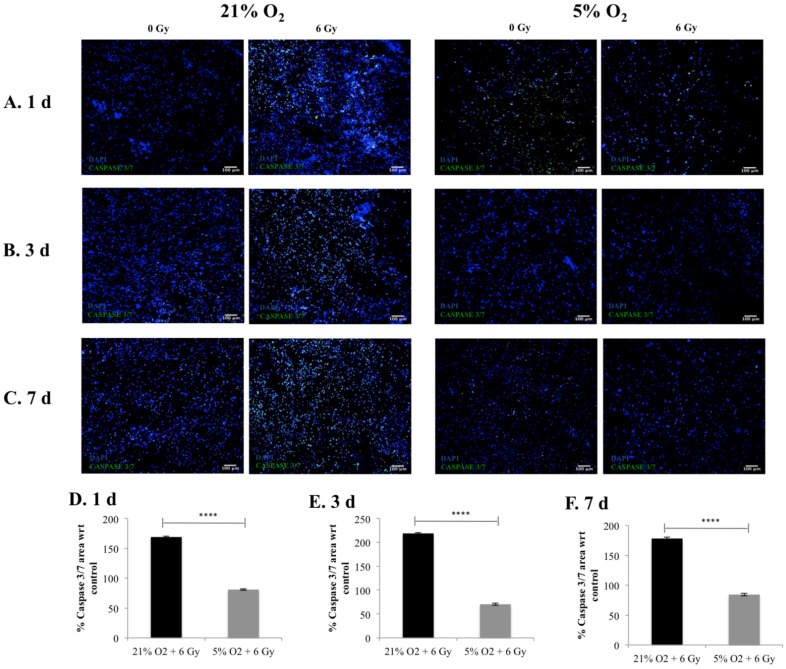
Apoptotic assay (Caspase 3/7) following radiotherapy treatment (6 Gy) in 3D scaffolds for 21% O_2_ and 5% O_2_: (**A**–**C**) Representative images of scaffold sections for Caspase 3/7 (green) and DAPI (blue) staining 1 day, 3 days, and 7 days post-treatment. (**D**–**F**) Equivalent image analysis based quantification of the percentage of Caspase 3/7 (green) image areas for (**A**–**C**) with respect to the control (untreated scaffolds). Multiple scaffolds (3), scaffold sections (3), and images (2) were analysed, mean values are presented. (**** = *p* < 0.0001).

Figure 2 and Figure 3 summarise the confocal images of scaffold sections showing the spatial distribution of Live/Dead and apoptotic cell areas, respectively, along with a quantitative assessment of the Live/Dead and apoptotic areas in multiple images taken from multiple sections of multiple scaffolds. Our results show a systematic trend of hypoxia-associated radio-protection of PANC-1 cells in the 3D scaffolds. More specifically, a significantly lower cell viability in the 3D scaffolds was seen at 21% O_2_ post-radiation treatment compared to 5% O_2_, where no statistical difference was observed between the treated and untreated 3D scaffolds at one, three, and seven days post-treatment (Figure 2). Similar results/trends were observed for apoptosis. More specifically, significantly higher apoptosis responses of the 3D scaffolds were observed at 21% O_2_ compared to 5% O_2_ at one, three, and seven days post radiation treatment (Figure 3). It should be highlighted that the cell viability (Figure 2) and cell apoptosis (Figure 3) trends in the 3D scaffolds were in line with the increase in the HIF-1a hypoxic biomarker (Figure 1), which showed a hypoxia associated radioprotection in the 3D scaffolds.

### 3.4. Post-Radiotherapy Monitoring of Collagen-I Expression in the 3D Scaffolds

**Figure 4:**
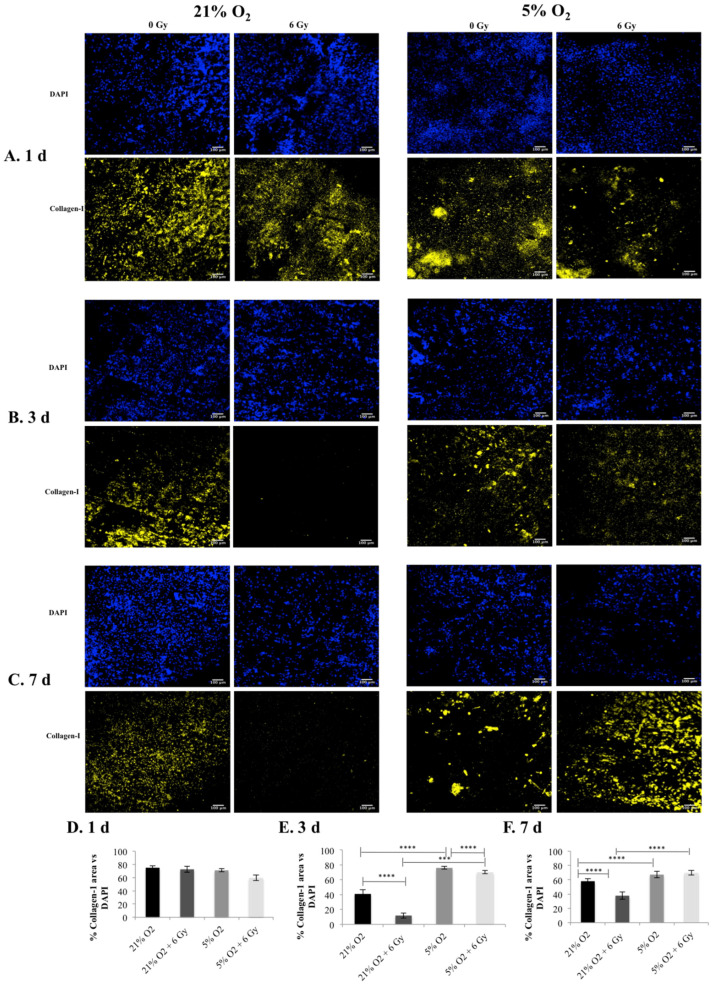
Collagen-I expression in 3D scaffolds, following radiotherapy treatment (6 Gy) for 21% O_2_ and 5% O_2_: (**A**–**C**) Representative images of scaffold sections for Collagen-I staining (yellow) and DAPI (blue) staining. (**D**–**F**) Equivalent image analysis based quantification of the percentage of collagen-I (yellow) areas for (**A**–**C**). Multiple scaffolds (3), scaffold sections (3), and images (2) were analysed, mean values were used. (**** = *p* < 0.0001). (*** = *p* < 0.001).

The expression of collagen-I (i.e., an ECM protein that is abundant in the PDAC tumour microenvironment and secreted by both stroma and cancer cells [55,62,63]) was monitored in the 3D scaffolds for all conditions under study. Figure 4 shows the immuno-fluorescence images of scaffold sections along with image quantification of the percentage of collagen-I positive areas for all conditions under study (Figure 4). Overall, we observed (i) higher collagen-I deposition in 5% O_2_ culture compared to 21% O_2_ culture for both the non-treated controls and the radiation treated samples at day 3 and 7, and (ii) the radiation treatment led to a substantially lower collagen-I secretion in 21% O_2_ compared to the respective control (untreated sample in 21% O_2_), while there was less collagen-I disruption in 5% O_2_ compared to the respective control at day 3 and at day 7 post-radiation treatment (Figure 4).

## 4. Discussion

In this work, we investigated, for the first time, the impact of in vitro hypoxia (5% O_2_) on the radiation treatment response of pancreatic cancer cells (PANC-1) in our recently developed polymer (polyurethane) based highly macro-porous 3D scaffold, which is surface modified with proteins (fibronectin) for better ECM mimicry [27]. The scaffold supports the long-term (37 days) culture of PDAC cells with cancer cell proliferation distribution in the scaffold similar to the one reported for mouse models for this time-frame, without requiring cell re-suspension, which would affect the established cell–cell, cell–matrix interactions and metabolic gradients [27,64].

In the current work, PANC-1 cells were seeded in polymeric scaffolds and were cultured for four weeks in in vitro normoxia (21% O_2_) followed by two days exposure to either in vitro hypoxia (5% O_2_) or maintenance in in vitro normoxia. Thereafter, radiation treatment followed by in situ post-radiation monitoring (Appendix A Figure A1) (one day, three days, seven days post-treatment) of the 3D cell cultures took place via quantification of (i) HIF-1a secretion by the cancer cells; (ii) live/dead and apoptotic profiles; and (iii) ECM (collagen-I) secretion by the cancer cells. For radiation treatment, a dose of 6 Gy was selected based on our previously published radiotherapy screening experiments in in vitro normoxia (i.e., 6 Gy was the dose we have shown to cause a reduction in cell viability but not total death in 21% O_2_ in our 3D scaffolds) [25]. In terms of the selection of the oxygen profile to create an in vitro hypoxic environment, 5% O_2_ was selected, in line with the literature as most papers reported hypoxia studies at oxygen ranges from 0.1% to 10% [47]. We refer to 5% O_2_ as in vitro hypoxia compared to 21% O_2_, which is in vitro normoxia and the oxygen level for which the PANC-1 cells are established and authenticated.

We report a reduction in PANC-1 sensitivity to ionising radiation, associated with in vitro hypoxia (i.e., increased cell viability (live cell profiles) (Figure 2)) and decreased cell apoptosis (Caspase 3/7 profiles) (Figure 3) trends in the 3D scaffolds, in-line with the increase in the HIF-1a hypoxic biomarker (Figure 1). This indicates HIF-1a (hypoxia induced) associated radioprotection. Moreover, in line with increases in HIF-1a, we report (i) higher collagen-I deposition in hypoxia and (ii) less collagen-I disruption post-irradiation treatment in hypoxic cultures at three days and seven days post-treatment (Figure 4). To the best of our knowledge, this research is the first study to demonstrate the feasibility of using a complex biomaterial-based scaffold for radiation response studies of PDAC, specifically addressing the hypoxia factor. Furthermore, most in vitro treatment screening studies for PDAC have a maximum timeframe of one-week culture and generally have a hypoxic exposure time of up to 72 h [28,65], while in this study, the total culture frame was five weeks (i.e., four weeks prior to treatment followed by a week of post-treatment monitoring (up to nine days exposure to hypoxia)).

As previously mentioned, there are a limited number of studies investigating the treatment of radiation in 3D PDAC models. For example, Hehlgans et al. (2009) utilised a spheroid 3D model for the pancreatic cancer cell line MiaPacCa2 treated with 0–6 Gy of irradiation to identify Caveolin-1 and TAE226 as potential radiosensitisers using a two day post treatment timeframe. Similar to our work, this model facilitates a post treatment analysis, however, the 3D model is spatially and structurally different to our scaffold (i.e., it is a spheroid), and the timeframe is different to our model, where we report a more extended post-treatment timeframe of one day, three days, and seven days post treatment [51,52]. Similar to our study, Al-Assar et al. (2014) reported radio-resistance in their in vitro sphere model of pancreatic cancer, however, the radio-resistance reported was associated with the co-culture of pancreatic stellate cells with pancreatic cancer cells and the study did not evaluate the role of hypoxia. More specifically, sphere models of the pancreatic cancer cell line PANC-1 and pancreatic stellate cell line PSC were cultured for a total timeframe of eight days and treated with 0–6 Gy to identify co-culture enhancement of radio-resistance [50]. Furthermore, similar to our work, Al-Ramadan et al. (2018) employed a 7-day post treatment Caspase assay to pancreatic cancer spheroids (BON-1) (12 day total culture) to identify radiation dose (0–6 Gy) (treatment at five days after seeding) [49].

More recently, Görte et al. (2020) investigated 3D PDAC spheroids and found that proton (low-LET of 3.7 keV/μm) irradiation stimulated higher efficacy to tumouroid formation and greater phosphoproteome alterations compared to conventional photon (200-kVp X-ray) irradiation in MiaPacCa-2, Capan-1, Panc-1, Patu8902, and COLO357 based spheroids [53]. Moreover, these 3D PDAC models were treated with 2, 4 or 6 Gy of photons or protons 24 h after seeding, thereafter 7–13 days (cell dependent) of incubation was given before post radiation analysis [53]. This total culture time differed to the 37-day total scaffold culture time frame described here. Similar to Görte et al. (2020), Yu et al. (2021) investigated new modalities for PDAC, describing PANC-1 and BxPC-3 spheroids co-cultured with fibroblasts for the investigation of boron neutron capture therapy (1.2 MW for 28 min) [54]. This research reported lower survival rates and higher apoptosis rates in 3D spheroids compared to 2D cultures after thermal neutron irradiation treatment [54].

Gupta et al. (2019) were the first to investigate polymeric scaffolds as a treatment-screening platform for chemo-radiotherapy [28]. The studies in 3D models for PDAC radiation treatment described did not incorporate the treatment limiting hypoxic hallmark of PDAC, in fact, studies reporting the combination of hypoxia and radiation for 3D cancer models in general are very limited in the literature. For example, similar to our observations, Indovina et al. (2006) reported an over-expression of HIF-1a associated with inhibition of radiation (2 Gy, 5 Gy) effects for a shorter time period (48 h) for human osteosarcoma spheroids [66]. Similarly to this, Simon et al. (2016) report a lung cancer cell based Cells-in-Gels-in-Paper 3D model (in which polyvinyl chloride multilayer sheets simulate a poorly vascularised tumour), identifying a HIF-1a over-expression and reduced sensitivity to ionising radiation when the distance between cells and oxygenated medium was increased, suggesting low oxygen association with radioprotection [67]. Despite the very limited literature available on investigating the role of hypoxia in 3D PDAC and other (in vitro) cancer models, hypoxia related radio-protection has been extensively reported in clinic [68] as well as in both animal models [69,70,71] and 2D in vitro systems [69,70,72].

As previously mentioned, we also reported a correlation between increases in HIF-1a levels in our 3D scaffolds and (i) higher collagen-I deposition in hypoxic cultures and (ii) less collagen-I disruption post-radiation treatment in hypoxic cultures. Studies of tumour hypoxia have revealed not only the impact of hypoxia of pancreatic cancer progression and invasion, but the stimulation of fibrosis and angiogenesis including collagen-I expression [55,62,73,74,75,76]. As an abundant and important protein of ECM, the expression of collagen-I is also reported to be linked to pancreatic cancer cell survival and progression [62,73,74]. Moreover, enhanced radio-resistance in pancreatic cancer cells has been identified in cells grown in the presence of ECM proteins (fibronectin) in 2D [75].

Generally, HIF-1a overexpression and collagen-I deposition correlation similar to that reported here, have mainly been described in 2D cell cultures, mouse models, and tissue samples in other cancer cell types. For example, HIF-1a activity is reported to promote ECM remodelling by inducing collagen-I expression in hypoxic fibroblast cultures [77]. Moreover, the study of liver fibrosis models revealed that HIF-1a deficient mouse models have shown reduced collagen-I and a-SMA levels (liver fibrosis) [78], suggesting hypoxia influence of collagen-I (ECM) secretion. Furthermore, collagen-I gene overexpression in line with lactate dehydrogenase (which aids glycolysis in hypoxic conditions) has been identified in radio-resistant cervical cancer tissue samples [79]. In recent years, there is a growing understanding of the importance of the ECM as a key player in treatment resistance and success, particularly in the complex pancreatic cancer TME (dense desmoplasia) [9,10,55,80,81]. Generally, the ability to map collagen-I deposition along with the response to in vitro hypoxia and radiation treatment is therefore an important aspect for the accuracy of a PDAC 3D model. Moreover, the biomimicry of cancerous tissues to develop models that accommodate the hypoxic and collagen rich environment to emulate physiological ecosystems is relevant for cancers that are renowned to be radio-resistant.

Overall, in this work, we describe a hypoxic PDAC 3D polymeric scaffold model for radiation treatment screening. We report increased levels of HIF-1a and collagen-I deposition in line with reduced PANC-1 sensitivity to ionising radiation in in vitro hypoxia. These cellular responses are characteristic of in vivo tumours. To the best of our knowledge, our work is the first to report long-term hypoxic PDAC culture and radiation treatment in biochemically and structurally complex 3D scaffolds. Generally, 3D hypoxic tissue research has been employed to understand the hypoxic behaviour of cells including cell migration and epithelial to mesenchymal transition [82,83,84]. However, there is a lack of 3D models investing the combination of hypoxia and radiotherapy treatment, despite the important role that hypoxia plays in radiotherapy treatment resistance. Therefore, developing structurally and biochemically complex models could accelerate our understanding of the link between radiotherapy treatment and spatial tissue characteristics, accelerating better therapies from bench to practice.

## 5. Conclusions

Overall, this work performed in vitro hypoxic radiation treatment screening on our recently published scaffold based PDAC model [27,28]. PANC-1 in vitro normoxia (21% O_2_) scaffolds were cultured for four weeks and then exposed to in vitro hypoxia (5% O_2_) or maintained in normoxic conditions (2 days), followed by radiation treatment (6 Gy). Thereafter, in situ post-radiotherapy monitoring (one day, three days, and seven days post-treatment) of the 3D cell cultures via quantification of (i) HIF-1a secretion by the cancer cells; (ii) Live/Dead and apoptotic profiles, and (iii) ECM (collagen-I) secretion by the cancer cells took place. Our analysis revealed increased levels of HIF-1a (Figure 1) in line with trends in increased cell viability (live cell profiles) (Figure 2) and decreases in cell apoptosis (Caspase 3/7 profiles) (Figure 3), which indicates HIF-1a (hypoxia) associated radioprotection. Moreover, we also report (in line with increases in HIF-1a) higher collagen-I deposition in in vitro hypoxic cultures and also less collagen-I disruption in in vitro hypoxic radiation treatment.

To the best of our knowledge, this is the first study to report an in vitro hypoxic PDAC long-term structurally and biochemically complex polymer based scaffold culture for radiation response studies and the first to correlate HIF-1a with increased collagen deposition post-radiation in a 3D PDAC model under hypoxia. Our system holds potential as an animal free alternative for predictive radiation research, providing more advanced spatial features compared to simple in vitro 2D models. Future work will focus on the investigation of the role of in vitro hypoxia on the PDAC cellular response to advanced radiotherapy modalities such as proton therapy and image guided radiotherapy using MR-Linacs in our complex multicellular scaffolds [36]. Furthermore, future research should also be performed in 3D systems with exposure to lower partial pressures of oxygen for even more physiologically relevant in vitro hypoxia.

## Data Availability

The datasets generated for this study are available on reasonable request to the corresponding author.
